# High prevalence and molecular spectrum of *β*-thalassemia trait in Haripur and Abbottabad districts of Pakistan: implications for regional carrier screening programs

**DOI:** 10.1186/s43042-025-00795-4

**Published:** 2025-10-15

**Authors:** Ehtisham ul Haq, Asma Naeem, Usman Ayub Awan, Sadiq Noor Khan, Muhammad Zohaib, Ghufran Uddin

**Affiliations:** 1 Department of Medical Lab Technology, Faculty of Biological & Biomedical Sciences, The University of Haripur, Haripur, 22600 Khyber Pakhtunkhwa Pakistan; 2 Alkhidmat Diagnostics, Karachi, 74600 Sindh Pakistan

**Keywords:** *β*-thalassemia trait, Molecular epidemiology, ARMS-PCR, Pakistan, Carrier frequency, Public-health genetics

## Abstract

**Background:**

*β*-thalassemia is a common autosomal recessive blood disorder caused by mutations in the *β*-globin gene, with highly variable carrier frequencies across different regions. In Pakistan, cultural practices such as consanguineous marriages, combined with limited access to genetic screening, contribute to an increased prevalence of inherited disorders. However, reliable data on community-level prevalence and molecular mutation patterns remain scarce, particularly in rural or semi-urban districts. This study aimed to investigate the prevalence and mutational spectrum of *β*-thalassemia trait in Haripur and Abbottabad districts of northern Pakistan to better inform prevention strategies.

**Results:**

Among 228 community participants, 28% (*n* = 64) were confirmed carriers (95% CI: 22–34%). ARMS-PCR screening of five common HBB mutations revealed that 59% (*n* = 38) carried IVS I-5 (G → C), 23% (*n* = 15) carried the FSC 8/9 (+ G) mutation, and 17% (*n* = 11) were compound heterozygotes for both, representing a noteworthy level of mutational complexity. No cases of IVS I-1 (G → T), Cd 41/42 (–TTCT), or the 619-bp deletion were detected. Carrier prevalence was significantly higher in Abbottabad than Haripur (42.9% vs. 13.2%; *p* <0.0001).

**Conclusion:**

This high local carrier rate, well above Pakistan’s national estimate of 5–8%, and the presence of compound heterozygotes underscore a concentrated genetic burden and the need for region-specific screening, premarital counseling, and expansion of molecular diagnostic services.

## Introduction

Thalassemia is one of the most prevalent monogenic disorders worldwide, with a particularly high burden in low- and middle-income countries. Globally, an estimated 1.5% of the population, about 80–90 million people, are carriers of *β*-thalassemia mutations, with regional carrier rates ranging from 5–20% in the Mediterranean, Middle East, and South Asia to less than 1% in most Northern European and North American populations [1]. Among its various forms, *β*-thalassemia is the most common and clinically significant, resulting from reduced or absent synthesis of the *β*-globin chain of hemoglobin due to mutations in the hemoglobin subunit beta** (**HBB) gene [2]. It follows an autosomal recessive inheritance pattern and contributes substantially to morbidity and healthcare costs in high-prevalence regions. Pakistan ranks among the countries most affected by thalassemia, with an estimated 100,000 individuals living with *β*-thalassemia major who require lifelong blood transfusions [3]. The carrier frequency of *β*-thalassemia in Pakistan is estimated at 5–8%, markedly higher than the global average of approximately 3%, translating to nearly 9.8 million carriers nationwide [4]. Each year, an estimated 5,000 children are born with *β*-thalassemia major, underscoring the urgent need for effective prevention and control strategies.

Despite being a preventable condition, thalassemia management in Pakistan remains largely palliative, relying on regular blood transfusions, iron chelation therapy, and supportive treatment for iron overload complications [5]. Curative approaches such as bone marrow transplantation are limited by cost, donor availability, and healthcare infrastructure. In this context, carrier detection, molecular screening, genetic counseling, and prenatal diagnosis (PND) represent critical components of any national prevention program. However, access to PND remains restricted to tertiary urban centers and is often constrained by low literacy rates, socioeconomic barriers, and limited healthcare coverage in rural areas [6]. Furthermore, the invasive nature of current PND procedures presents additional ethical and medical challenges.

The molecular basis of *β*-thalassemia in Pakistan is relatively well-characterized, with five mutations including IVS I-5 (G → C), FSC 8/9 (+ G), Cd 41/42 (–TTCT), 619 bp deletion, and IVS I-1 (G → T) accounting for over 90% of mutant alleles [2]. Nationally, IVS I-5 (G → C) and FSC 8/9 (+ G) are the two most frequently reported *β*-thalassemia mutations, but their relative frequencies vary geographically. For example, IVS I-5 predominates in Punjab and Khyber Pakhtunkhwa, whereas FSC 8/9 is more common in southern districts such as Kohat. Neighboring countries show similar heterogeneity: Iranian cohorts report IVS I-5 and IVS I-1 as major mutations, while Indian populations often carry Cd 41/42 deletions [1, 7, 8]. These differences underscore the importance of region-specific molecular data for effective screening strategies. The high prevalence of consanguineous marriages in Pakistan exacerbates the risk of homozygosity, with studies reporting sibling carrier rates as high as 61% in affected families [9, 10]. However, most epidemiological studies conducted thus far have been restricted to hospital-based or tertiary care settings in major urban centers of Pakistan. This limited scope may lead to an underestimation of the true community-level prevalence of *β*-thalassemia carriers. Furthermore, such studies fail to account for regional variations in mutation frequency, consanguinity patterns, and public awareness, particularly in rural and semi-urban populations.

Despite Pakistan’s high overall carrier rate, molecular data from the northern districts of Haripur and Abbottabad are scarce, and no population-based studies have systematically assessed the local mutation spectrum. To address this gap, the present study investigates the molecular epidemiology of the *β*-thalassemia trait in the Haripur and Abbottabad districts of northern Pakistan through a community-based household survey. Using hematological screening, hemoglobin electrophoresis, and ARMS-PCR for common *β*-thalassemia mutations, this study aims to provide reliable prevalence data and mutation spectrum from a general population cohort in this region. The detection of compound heterozygotes further underscores the unexpected genetic complexity. The findings are intended to inform regional and national thalassemia control policies by highlighting the magnitude of silent carrier burden and the need for widespread genetic screening initiatives.

## Materials and methods

### Study setting and design

This cross-sectional study was conducted over a 12-month period at the University of Haripur, with community-based sample collection in the Haripur and Abbottabad districts of Khyber Pakhtunkhwa, Pakistan. Collected samples were transported to Department of Medical Lab Technology at the University of Haripur for hematological and molecular analyses, including complete blood counts (CBC), peripheral blood smear examination, hemoglobin electrophoresis, DNA extraction and quantification, and PCR amplification.

### Sampling strategy

District Haripur comprises three tehsils including Ghazi, Haripur, and Khanpur distributed across 155 village councils with a population of approximately 1.003 million. District Abbottabad includes two tehsils Abbottabad and Havelian encompassing 195 village councils and a population of around 1.332 million [11]. A multistage stratified random sampling approach was employed. In the first stage, 10% of village councils from each tehsil were selected at random, creating five geographic strata. In the second stage, 46 households were randomly selected from each stratum, and one individual per household was enrolled, irrespective of age or gender. The sample size (*n* = 228) was calculated using the standard formula for prevalence studies:

$$n = \frac{{Z^{2} P \left( {1 - P} \right)}}{{d^{2} }}$$

where *Z* = 2.576 (for 99% confidence level), *P* = 0.08 (estimated prevalence of *β*-thalassemia in Pakistan), *d* = 0.05 (margin of error). Substituting these values:

$$n = \frac{{\left( {2.576} \right)^{2} \times 0.08 \times 0.92}}{{(0.05)^{2} }} \approx { }196$$

To allow for potential non-response and sample exclusions, we inflated this target by ~ 15% (196 × 1.15 ≈ 226) and therefore recruited 228 participants. A structured questionnaire, adapted from previously published thalassemia awareness surveys and reviewed by two public-health experts for face validity, was used to collect sociodemographic data, family history of genetic disorders, and awareness regarding thalassemia. The questionnaire was interviewer-administered in the local language by trained field staff. Written informed consent was obtained from all participants. The study was approved by the institutional ethics review committee (Ref No: UOH/DASR/3080).

### Inclusion and exclusion criteria

Individuals aged 5–60 years and with permanent residency in the Haripur or Abbottabad districts were eligible for inclusion. Individuals with a known diagnosis of thalassemia or other hemoglobinopathies, children under five years (to avoid inclusion of homozygotes), and individuals over 60 years (due to logistical and ethical considerations) were excluded.

### Hematological analysis

A 5 mL venous blood sample was collected from each participant using EDTA-coated vacutainers (Xinle, China), following Clinical and Laboratory Standards Institute (CLSI) guidelines [12]. CBC was performed using an automated hematology analyzer (Sysmex XP-100, Japan), and peripheral blood smears were stained with Giemsa (Merck, Germany) to assess red blood cell morphology. Samples showing microcytic hypochromic indices, specifically MCV ≤ 76 fL and/or MCH ≤ 27 pg and the presence of target cells (codocytes), were further analyzed by high performance liquid chromatography (HPLC) for hemoglobin profiling (Bio-Rad, USA). A HbA_2_ level > 3.5% was considered diagnostic for *β*-thalassemia trait [13].

### Molecular analysis

Genomic DNA was extracted from peripheral leukocytes using the GeneJET Genomic DNA Purification Kit (Thermo Scientific, USA) according to the manufacturer’s instructions. Mutation analysis was conducted using allele-specific amplification refractory mutation system-PCR (ARMS-PCR) to detect five common *β*-thalassemia mutations prevalent in Pakistan including IVS I-5 (G–C), FSC 8/9 (+ G), Cd 41/42 (–TTCT), IVS I-1 (G → T), and 619 bp deletion. Primer sequences and PCR conditions (Table 1) were adopted from previously published protocols [8]. Each ARMS-PCR reaction (20 µL) consisted of 10 µL EasyTaq PCR SuperMix (TransGen Biotech), 0.5 µL each of allele-specific and control primer (internal controls A and B, common primer C, and mutant/normal allele-specific primer—see Table 1), 1 µL genomic DNA (~ 10 ng) and nuclease-free water to 20 µL. Thermal cycling conditions were: initial denaturation at 94 °C for 2 min, followed by 25 cycles of denaturation at 94 °C for 1 min, primer-specific annealing for 30 s, extension at 72 °C for 1 min, and a final extension at 72 °C for 6 min. PCR products were resolved on 2% agarose gel electrophoresis containing ethidium bromide, run at 110 V for 35 min (Bio-Rad, USA), and visualized using a gel documentation system (Labtron, UK). Expected fragment sizes for the internal control and mutation-specific amplicons are listed in Table 1. The presence of a mutation-specific band together with the internal control band was interpreted as positive for the targeted allele (heterozygote/trait). Samples with only the internal control band and no mutation-specific band were interpreted as negative for that mutation. For the 619 bp deletion assay, the absence/presence of a diagnostic amplicon was interpreted according to the published assay design.

**Table 1:** Optimized conditions for ARMS-PCR primers

Mutation	Primer	Direction	Sequence (5′ → 3′)	Length (bp)	Tm (°C)	GC%	Optimized Tm (°C)	Product Size (bp)
IVS 1–5 (G–C)	Mt	Reverse	CTCCTTAAACCTGTCTTGTAACCTTGATAG	30	59	40	61	293
	N	Reverse	CTCCTTAAACCTGTCTTGTAACCTTGATAC	30	59	40		
FSC 8/9 (+ G)	Mt	Reverse	CCTTGCCCCACAGGGCAGTAACGGCACACC	30	73	67	63	222
	N	Reverse	CCTTGCCCCACAGGGCAGTAACGGCACACT	30	73	63		
Cd 41/42 (–TTCT)	Mt	Reverse	GAGTGGACAGATCCCCAAAGGACCAACCT	29	67	55	65	451
	N	Reverse	GAGTGGACAGATCCCCAAAGGACTCAAAG	29	64	52		
IVS I-1 (G → T)	Mt	Reverse	TTAAACCTGTCTTGTAACCTTGATACGAAA	30	59	33	62	289
	N	Reverse	TTAAACCTGTCTTGTAACCTTGATACGAAC	30	59	37		
Common C	Common C	Forward	TCACTTAGACCTCACCCTGTGGAGCCA	27	66	56	–	–
Internal Control	Control A	Forward	CAATGTATCATGCCTCTTTGCACC	24	58	46	63	862
	Control B	Reverse	GAGTCAAGGCTGAGAGATGCAGGA	24	62	54		

*Bp* base pair, *Tm* melting temperature, *GC* Guanine cytosine

### Statistical analysis

Data were analyzed using R software version 3.3.2. Descriptive statistics were applied to obtain means, medians, and frequencies of demographic and clinical variables. Inferential analysis involved the Pearson’s Chi-squared test, Wilcoxon rank-sum test, and Fisher’s exact test, as appropriate. The significance level of *p* < 0.05 was considered statistically significant.

## Results

### Study area and participant demographics

**Fig. 1:**
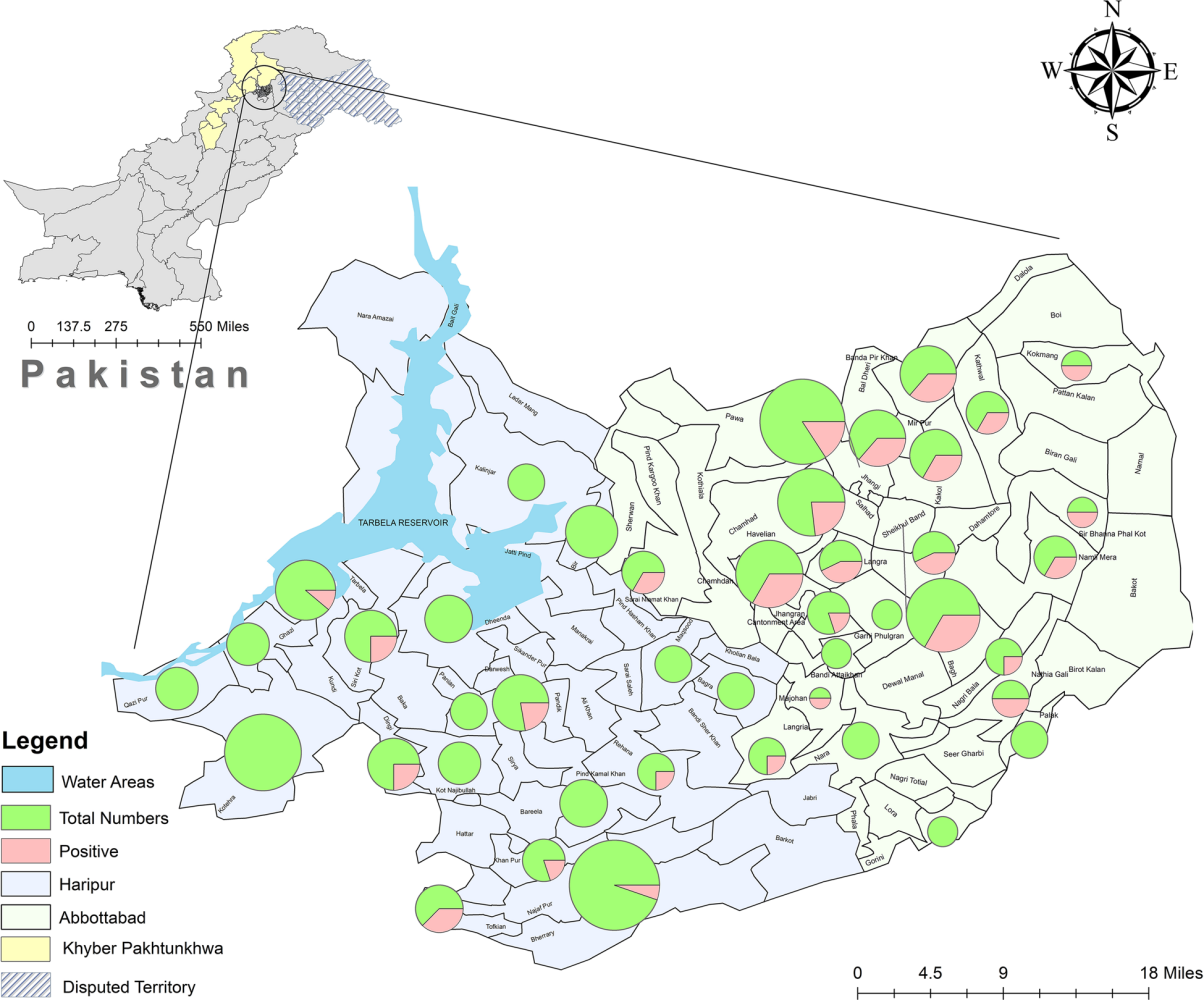
Geographic distribution and prevalence of *β*-thalassemia trait in the districts of Haripur and Abbottabad. The map illustrates spatial variation in carrier frequency across village councils using color shading. Pie charts represent the number of individuals sampled and the proportion testing positive for the *β*-thalassemia trait at each location

A total of 228 individuals were sampled from various village councils across the Haripur and Abbottabad districts. Of the 228 participants, 64 were *β*-thalassemia carriers, giving a prevalence of 28.1% (95% CI 21.9–34.9). Carrier prevalence was significantly higher (χ² = 23.7, *p* <0.0001) in Abbottabad (42.9%, 95% CI 33.9–52.4) compared to Haripur district (13.2%,95%, CI 7.6–20.8) . The geographic distribution of sampling locations is illustrated in Fig. 1, which displays regional differences in *β*-thalassemia trait prevalence through color-coded shading and pie charts representing sample sizes and trait positivity rates per location. Among the total participants, 57% (*n* = 129) were male, among whom 21% (*n* = 27) were positive for the *β*-thalassemia trait. In contrast, 43% (*n* = 99) were female, with a higher positivity rate of 37% (*n* = 37). Participant ages ranged from 15 to 58 years, with a mean age of 30 years. The sample included individuals from both rural (53%, *n* = 120) and urban (47%, *n* = 108) settings. Regional differences in trait prevalence were observed: The Haripur district showed a relatively lower frequency, with trait prevalence ranging from 5 to 11% across the Ghazi, Haripur, and Khanpur tehsils. In contrast, Abbottabad district exhibited substantially higher rates, with 59% prevalence in Abbottabad tehsil and 17% in Havelian. Further demographic details, including participants’ educational background, caste affiliations, parental relationship status, and village council-wise breakdown, are provided in Table 2.

**Table 2:** Demographic distribution and prevalence of beta thalassemia trait in District Haripur and Abbottabad

Characteristic	*N*	Negative, *N* = 164^*1*^	Positive, *N* = 64^*1*^	*p*-value^*2*^	df	X2 Value	Binary logistic regression					
							Coefficient ^*3*^	95% CI^*1*^	*p*-value	OR^*1*^	95% CI^*1*^	*p*-value
Gender	228			0.01	1	6.71						
Female		62 (38%)	37 (58%)				—	—		—	—	
Male		102 (62%)	27 (42%)				0.59	− 0.18, 1.4	0.14	1.8	0.83, 3.92	0.14
Age	228			0.6								
05–15		6 (3.7%)	1 (1.6%)				—	—		—	—	
16–25		66 (40%)	24 (38%)				− 1.2	− 4.3, 0.96	0.3	0.3	0.01, 2.61	0.3
26–35		43 (26%)	17 (27%)				− 1.3	− 4.5, 1.1	0.3	0.28	0.01, 2.96	0.3
36–45		32 (20%)	17 (27%)				− 1.4	− 4.6, 1.1	0.3	0.26	0.01, 2.96	0.3
46–55		12 (7.3%)	5 (7.8%)				− 1.6	− 5.0, 1.1	0.3	0.21	0.01, 3.06	0.3
56–60		5 (3.0%)	0 (0%)				NA	NA	NA	NA	NA	NA
Tehsil	228			< 0.001	4	28.4						
Abbottabad		42 (26%)	38 (59%)				—	—		—	—	
Ghazi		32 (20%)	3 (4.7%)				2.8	1.4, 4.5	< 0.001	15.9	3.87, 93.2	< 0.001
Haripur		40 (24%)	7 (11%)				1.7	0.64, 2.9	0.003	5.43	1.89, 17.6	0.003
Havelian		23 (14%)	11 (17%)				0.33	− 0.69, 1.4	0.5	1.39	0.50, 3.92	0.5
Khanpur		27 (16%)	5 (7.8%)				1.8	0.52, 3.3	0.01	5.84	1.69, 25.8	0.01
Origen	228			0.2	1	2.02						
Rural		81 (49%)	39 (61%)				—	—		—	—	
Urban		83 (51%)	25 (39%)				0.27	− 0.46, 1.0	0.5	1.31	0.63, 2.76	0.5
Mother tongue	228			0.12	2	4.24						
Hindko		101 (62%)	37 (58%)				—	—		—	—	
Pashto		25 (15%)	5 (7.8%)				− 0.71	− 2.1, 0.72	0.3	0.49	0.12, 2.05	0.3
Urdu		38 (23%)	22 (34%)				− 0.53	− 1.4, 0.32	0.2	0.59	0.25, 1.37	0.2
Education	228			0.3								
Advance		66 (40%)	18 (28%)				—	—		—	—	
Illiterate		7 (4.3%)	3 (4.7%)				− 0.13	− 2.6, 2.4	> 0.9	0.88	0.08, 11.6	> 0.9
Intermediate		81 (49%)	36 (56%)				− 0.88	− 2.5, 0.69	0.3	0.41	0.09, 2.00	0.3
Primary		10 (6.1%)	7 (11%)				− 0.97	− 3.1, 1.1	0.4	0.38	0.05, 3.04	0.4
Economic status	228			0.2								
High		6 (3.7%)	0 (0%)				—	—		—	—	
Low		45 (27%)	21 (33%)				− 18		> 0.9	0		> 0.9
Middle		113 (69%)	43 (67%)				− 17		> 0.9	0		> 0.9
Marital status	228			0.058	1	3.61						
Married		83 (51%)	42 (66%)				—	—		—	—	
Unmarried		81 (49%)	22 (34%)				0.52	− 0.51, 1.6	0.3	1.69	0.60, 4.93	0.3
Parental relation	228			0.5								
Cousin		94 (57%)	42 (66%)				—	—		—	—	
Out of family		63 (38%)	19 (30%)				0.46	− 0.33, 1.3	0.3	1.58	0.72, 3.57	0.3
Within biradari		7 (4.3%)	3 (4.7%)				1	− 0.57, 2.9	0.2	2.77	0.57, 18.2	0.2
Family history of thalassemia	228			0.003		9.83						
Nil		162 (99%)	57 (89%)				—	—		—	—	
Present		2 (1.2%)	7 (11%)				− 2.5	− 4.9, − 0.57	0.019	0.08	0.01, 0.57	0.019
Knowledge level about thalassemia	228			0.3	2	2.35						
High		33 (20%)	15 (23%)				—	—		—	—	
Moderate		41 (25%)	10 (16%)				0.22	− 1.0, 1.5	0.7	1.24	0.35, 4.30	0.7
Nil		90 (55%)	39 (61%)				0.67	− 0.96, 2.3	0.4	1.95	0.38, 10.1	0.4
Mutation type	64											
Dual		0 (NA%)	11 (17%)				NA					
FSC 8/9		0 (NA%)	15 (23%)				NA					
IVS I− 5		0 (NA%)	38 (59%)				NA					

^1^n (%), ^2^Pearson’s Chi-squared test, ^3^*OR* Odds Ratio, *CI* Confidence Interval, *NA* Not Analyzed

### Hematological findings

All blood samples underwent CBC analysis, followed by peripheral blood smear and hemoglobin electrophoresis. Four hematological parameters were evaluated for initial screening: MCH (< 27 pg), MCV (< 80 fL), RBC count (> 3.5 × 10^12^/L), and Hb (< 12 g/dL). Samples with low MCH in combination with at least two additional abnormal parameters were considered presumptive *β*-thalassemia trait cases. Based on this criterion, 69 out of 228 samples (30%) were flagged as suspected carriers. Peripheral blood smears of these 69 samples revealed varying degrees of anisocytosis: (+) in 8 cases, 28 with (+ +) in 28 cases, (+ + +) in 21 cases, and (+ + + +) in 10 cases. Additionally, target cells (codocytes) were identified in 50 of these 69 samples, along with prominent microcytic and hypochromic features. Although anisocytosis is suggestive of *β*-thalassemia trait, it is also present in iron deficiency anemia and must be interpreted in context. Confirmation was achieved using hemoglobin electrophoresis via HPLC, where 64 out of 228 samples (28%) showed diagnostic indicators of *β*-thalassemia trait. These included elevated HbA2 levels (> 3.5%), reduced HbA (< 94%), and elevated HbF (> 0.8%). These findings align with characteristic hematological profiles of *β*-thalassemia trait. Detailed comparisons of hematological parameters and their statistical significance are presented in Table 3, while Table 4 summarizes blood indices and hemoglobin fractions across detected genotypes.

**Table 3:** Mean value of hematological parameters and Hb electrophoresis in *β*-thalassemia trait and normal individuals

Hematological parameters	Normal individuals	*β*-thalassemia trait	P-value
RBC (10^6^/µl)	4.6 (4.2–5.0)	5.5 (4.7–6.2)	< 0.001
Hb (g/dl)	14.3 (13.0–15.2)	11.5 (10.2–12.4)	< 0.001
HCT (%)	39.0 (36.1–41.8)	35.9 (32.4–38.5)	< 0.001
MCV (fL)	84 (81–87)	63 (62–67)	< 0.001
MCH (pg)	30.5 (29.0–31.9)	20.2 (19.1–22.2)	< 0.001
MCHC (g/dl)	36.4 (35.5–37.0)	32.1 (31.4–33.1)	< 0.001
RDW-SD (fL)	39.5 (37.5–42.1)	37.6 (35.4–40.6)	< 0.001
RDW-CV (%)	13.0 (12.3–14.0)	17.7 (16.0–19.4)	< 0.001
RDW index	698 (634–852)	446 (366–575)	< 0.001
HbA (%)	96.5 (96.0–97.0)	94.0 (93.0–95.0)	< 0.001
HbF (%)	1.7 (1.0–7.0)−	4.2 (1.0–7.0)	> 0.9
HbA2 (%)	2.1 (2.0–2.3)	5.0 (4.2–5.1)	< 0.001

**Table 4:** Mean value of hematological parameters and Hb electrophoresis in various genotypes

Hematological parameters	*β*/*β* ^IVS I−5^	*β*/*β*^FSC8/9^	*β*^FSC8/9^/*β*^IVS I−5^	Normal individuals
Hb (g/dl)	10.9 (5.6–17.0)	12.1 (9.7–13.4)	11.2 (9.3–13.6)	14.3 (13.0–15.2)
RBC (10^6^/µl)	5.1 (3.14–6.7)	6.0 (4.5–6.8)	5.4 (4.0–6.4)	4.6 (4.2–5.0)
MCV (fL)	66.0 (56.7–77.6)	63.3 (57.9–73.8)	63.3 (57.5–77.1)	84 (81–87)
MCH (pg)	21.3 (14.0–27.9)	21.0 (18.2–31.9)	20.3 (18.1–26.7)	30.5 (29.0–31.9)
MCHC (g/dl)	32.1 (24.0–36.4)	32.1 (29.6 –35.2)	32.0 (30.0–34.6)	36.4 (35.5–37.0)
HbA (%)	93.5 (88.0–95.0)	93.9 (88.0–95.0)	93.1 (88.0–95.0)	96.5 (96.0–97.0)
HbF (%)	4.0 (0.8–7.0)	3.4 (0.8–7.0)	4.8 (1.0–7.0)	1.7 (1.0–7.0)
HbA2 (%)	4.8 (4.0–5.6)	4.7 (4.0–5.6)	4.7 (4.0–5.6)	2.1 (2.0–2.3)

### Molecular analysis

**Fig. 2:**
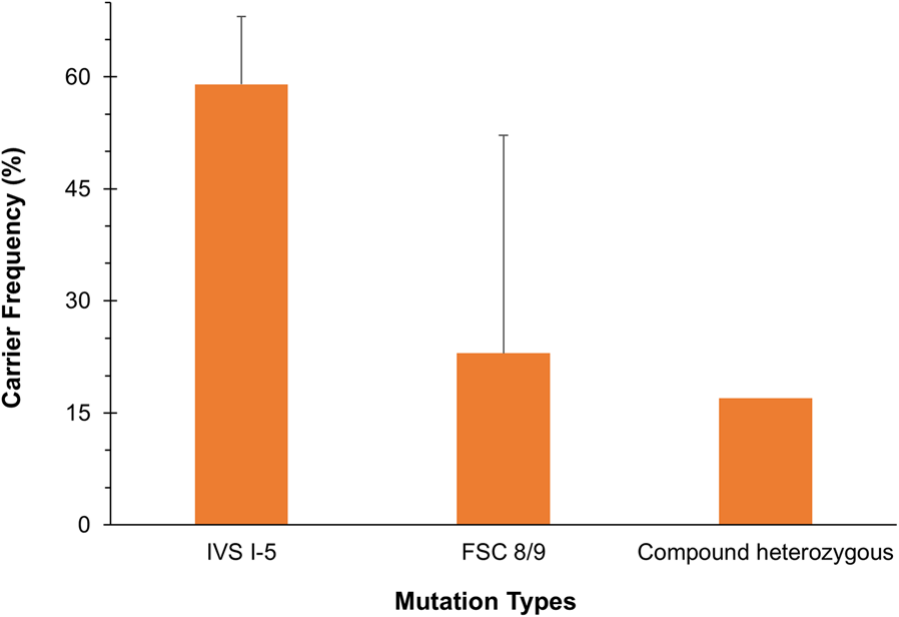
Frequency of *β*-thalassemia mutations among confirmed carriers in Haripur and Abbottabad districts (*n* = 64). Error bars indicate 95% confidence intervals for each estimate

**Fig. 3:**
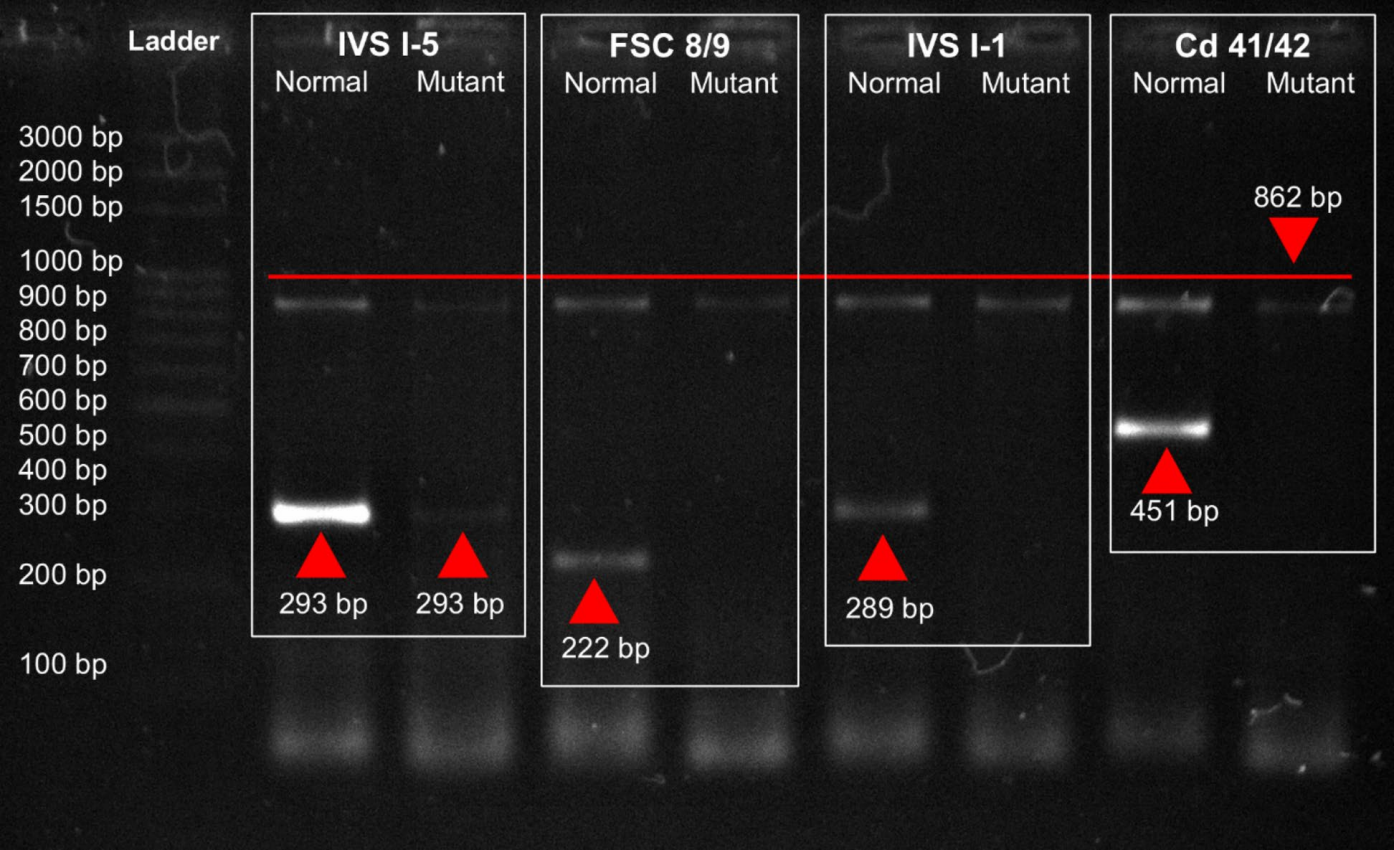
Detection of IVS I-5 mutation by ARMS-PCR. Representative ARMS-PCR gel image showing amplified fragments for the IVS I-5 (G → C) mutation. The presence of the 293 bp mutant band confirms the mutation, while the control fragment (862 bp) validates PCR success. The absence of amplification for the other mutation-specific primers indicates that these mutations are not present in the sample. A 3 kb DNA marker was used for size comparison of the amplified products

**Fig. 4:**
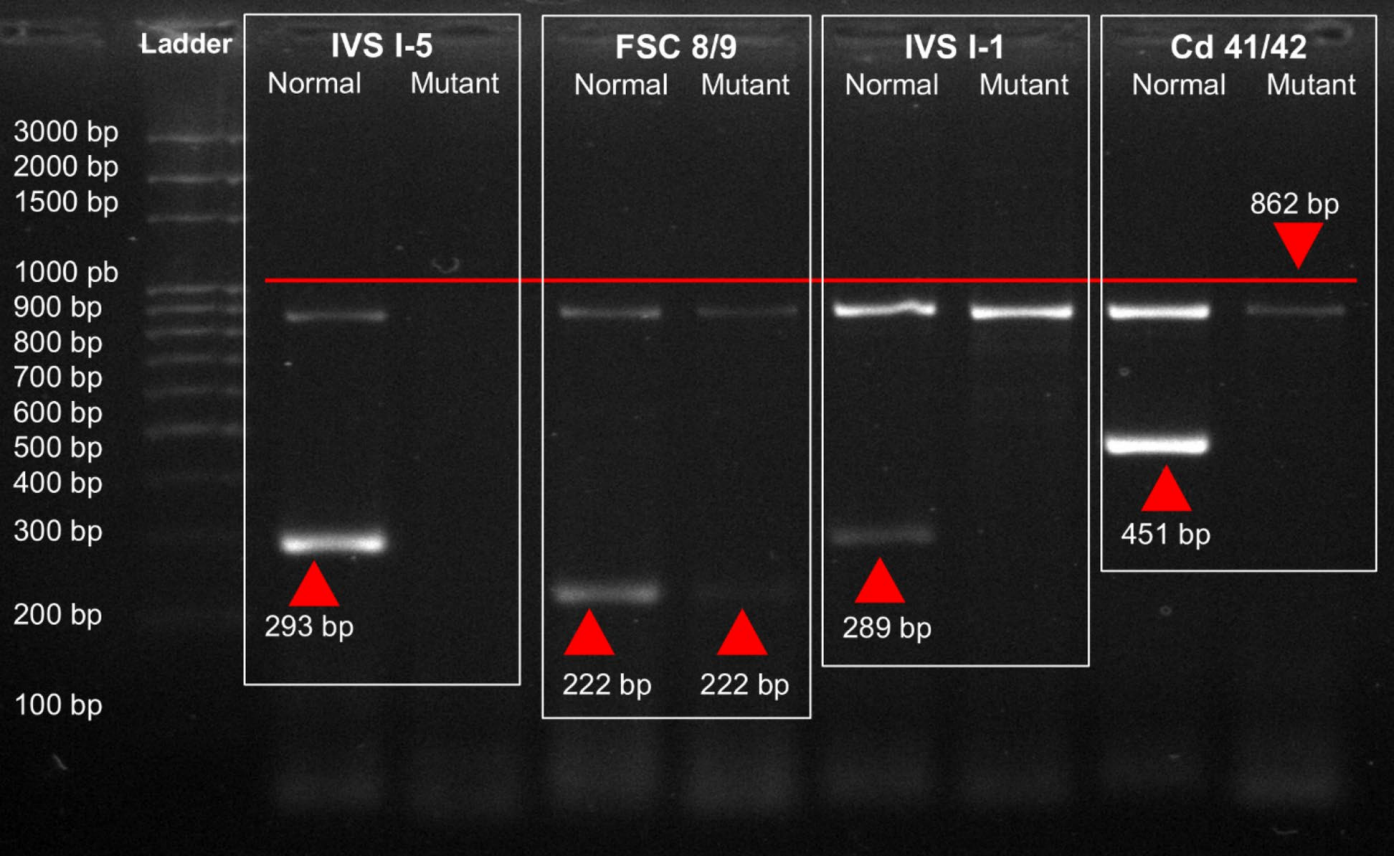
Detection of FSC 8/9 mutation by ARMS-PCR. Representative ARMS-PCR gel image showing amplified fragments for the FSC 8/9 (+ G) mutation. The presence of the 222 bp mutant band confirms the mutation, while the control fragment (861 bp) validates PCR success. The absence of bands for primers targeting other mutations confirms that these mutations are not present in the sample. A 3 kb DNA marker was used for size comparison of the amplified products

**Fig. 5:**
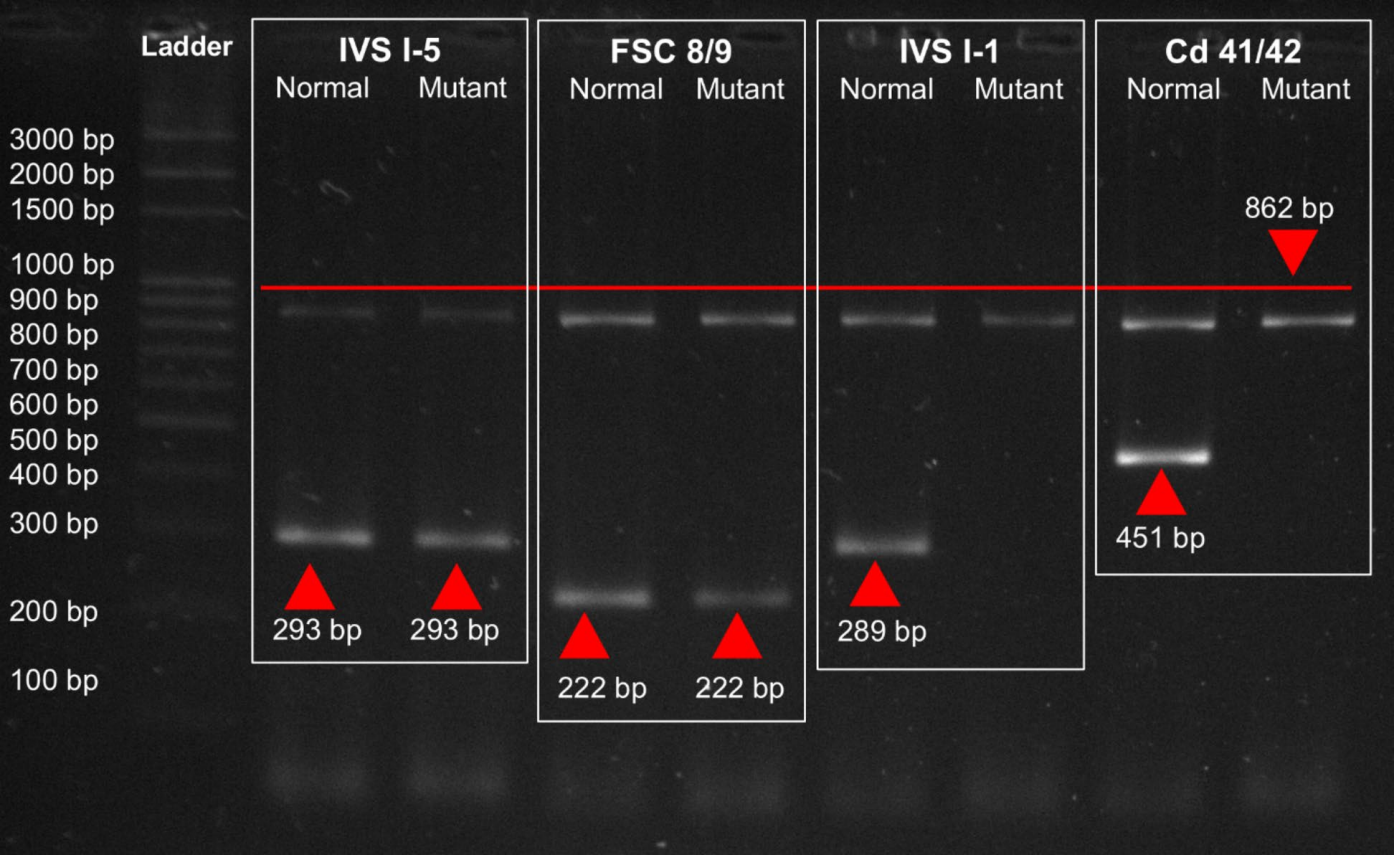
ARMS-PCR and gel electrophoresis analysis of a representative sample positive for compound heterozygosity, IVS I-5 and FSC 8/9. Control primers amplified an 862 bp fragment as an internal control. The common C forward primer, along with specific reverse primers for IVS I-5 and FSC 8/9 mutations, produced ARMS products of 293 bp and 222 bp, respectively. The presence of bands for both normal and mutant primers for each mutation confirms that the sample is compound heterozygous for IVS I-5 and FSC 8/9. The absence of bands for primers targeting other mutations confirms their non-presence in the sample. A 3 kb DNA marker was used for size

Genomic DNA was extracted from all 64 electrophoresis-confirmed *β*-thalassemia carriers and subjected to mutation analysis using the amplification refractory mutation system-PCR (ARMS-PCR), targeting five common *β*-thalassemia mutations frequently reported in the Pakistani population: IVS I-5 (G → C), FSC 8/9 (+ G), IVS I-1 (G → T), Cd 41/42 (–TTCT), and the 619 bp deletion. Among carriers, IVS I-5 accounted for 59% (n = 38, 95% CI 46.5–70.7), FSC 8/9 for 23% (n = 15, 95% CI 13.6–35.2), and 17% (n = 11, 95% CI 9.1–29.2) were compound heterozygotes. No IVS I-1, Cd 41/42, or 619 bp deletions were detected. These findings indicate that IVS I-5 is the predominant *β*-thalassemia mutation in this cohort (Fig. 2). Figures 3, 4, and 5 show ARMS-PCR and gel electrophoresis analysis of a representative sample positive for IVS I-5, FSC 8/9 and compound heterozygotes, respectively. The distribution of these mutations varied by district, with a notably higher concentration of IVS I-5 cases observed in Abbottabad compared to Haripur, as detailed in Table 5.

**Table 5:** Distribution of beta thalassemia mutations in Haripur and Abbottabad

Mutation	Total	Haripur district [*n* (%)]			Abbottabad district [*n* (%)]	
		Ghazi	Haripur	Khanpur	Abbottabad	Havelian
IVS I-5	38	0 (0)	5 (13)	3 (8)	24 (63)	6 (16)
FSC 8/9	15	3 (20)	1 (7)	1 (7)	7 (47)	3 (20)
Compound	11	0 (0)	1 (9)	1 (9)	7 (64)	2 (18)
IVS I-1	–	–	–	–	–	–
Cd 41/42	–	–	–	–	–	–
Total	64	3 (5)	7 (11)	5 (8)	38 (59)	11 (17)

## Discussion

Despite extensive research on *β*-thalassemia in Pakistan, most epidemiological studies have been conducted in hospital-based or tertiary care settings in major urban centers. This limited scope likely underestimates the true community-level burden and overlooks regional disparities in mutation frequency, consanguinity patterns, and genetic awareness, particularly in rural and semi-urban areas. Our cross-sectional study, focusing on household populations in Haripur and Abbottabad, reveals a strikingly high overall *β*-thalassemia carrier rate of 28%, with substantial inter-district variation. The 59% (38/64) carrier rate in Abbottabad stands in stark contrast to the 4.7% (3/64) observed in Tehsil Ghazi, Haripur (p < 0.001), emphasizing the importance of region-specific surveillance strategies. The prevalence identified in this study greatly exceeds national carrier estimates of 5–8% [3] and rates reported in earlier studies 5.6% in Azad Kashmir [14], 4% in Islamabad and Rawalpindi [15], and 5.5% in Karachi [13]. While higher prevalence has been documented in urban areas such as Faisalabad (44.4%) and Lahore (52%) [10, 21], our data from a largely rural population suggest that the carrier burden in peripheral regions may be similarly high but underreported. An earlier study from Abbottabad also found a 58.2% trait prevalence [16], corroborating our findings and indicating that localized genetic and social dynamics, particularly consanguinity, may be responsible for these elevated rates.

Consanguineous marriages, particularly cousin unions, remain common in northern Pakistan and have been shown to significantly increase the prevalence of autosomal recessive disorders, including *β*-thalassemia. In our study population, 64% of marriages were between blood relatives, primarily cousins, which likely contributes to the elevated carrier rates observed in both districts. Previous studies have consistently shown that consanguineous unions increase the likelihood of both parents carrying the same deleterious allele [17–19]. However, a critical finding of our study is that 89% of identified carriers were unaware of any family history of thalassemia, reflecting a lack of awareness even among genetically at-risk families. The statistically significant difference in family history between carriers and non-carriers (*p* = 0.003) suggests that awareness campaigns have failed to permeate rural populations, where cultural practices around marriage remain largely unchanged. Although the difference in trait prevalence between married (66%) and unmarried (34%) individuals was not statistically significant (*p* = 0.058), the high prevalence among married participants implies that uninformed marriages, especially in consanguineous contexts, remain a key challenge [20].

From a diagnostic perspective, microcytic hypochromic indices were found in 30% of our cohort, which aligns with previously reported rates ([21]; [22]). While this hematologic profile is consistent with *β*-thalassemia carriers, it overlaps with iron deficiency anemia, making sole reliance on CBC and peripheral smear analysis inadequate for definitive diagnosis. This reinforces the importance of confirmatory testing via Hb electrophoresis and, ideally, molecular diagnostics, which remain inaccessible in many rural areas. Our molecular findings show IVS I-5 (G → C) as the most prevalent mutation (59%), followed by FSC 8/9 (+ G) at 23%, both consistent with prior regional reports [7, 8]. The prevalence of IVS I-5 was particularly high in Abbottabad, suggesting a potential founder effect or regional clustering. Interestingly, 17% of samples exhibited compound heterozygosity for both IVS I-5 and FSC 8/9, an observation not previously reported in this region. While these individuals were phenotypically classified as carriers, the presence of dual mutations may influence clinical severity in future generations if inherited in homozygous or compound heterozygous states. Studies from neighbor countries including Iran and India have demonstrated that compound heterozygosity can produce atypical hematological profiles and affect clinical outcomes [23, 24]. The absence of other common mutations such as IVS I-1, Cd 41/42, and the 619 bp deletion may reflect regional mutation patterns or limitations in mutation panels used for ARMS-PCR screening.

Critically, the detection of dual mutations in 11 of 64 carriers raises important questions regarding the adequacy of current genetic screening frameworks, which often overlook compound genotypes. The absence of comprehensive prenatal diagnostic services and molecular characterization programs further limits early detection and genetic counseling. Given that dual mutations may remain undiagnosed without molecular testing, our findings call for expanded sequencing-based approaches, particularly in areas with high consanguinity and carrier burden. Awareness campaigns, compulsory premarital screening, and the integration of molecular diagnostics into primary healthcare are critical steps toward reducing the thalassemia burden. Without such measures, the prevalence of *β*-thalassemia carriers and affected births will continue to rise unchecked, especially in rural regions where genetic risks are compounded by sociocultural norms and health service inequities.

## Study limitations

This study has several limitations that should be considered when interpreting the findings. First, the mutation analysis focused on a panel of common variants, which may have missed rare or novel mutations due to the absence of comprehensive gene sequencing. ARMS-PCR is an allele-specific assay that provides high analytical specificity for the detection of predefined single-nucleotide and small insertion/deletion mutations when validated primers and appropriate controls are used. Its principal limitation is that it detects only the targeted mutations and will not identify novel or untargeted variants. Second, while individuals with dual mutations were identified, detailed clinical or hematological assessments were not performed, limiting our ability to evaluate their phenotypic severity or confirm compound heterozygosity. Additionally, family history and consanguinity data were self-reported and thus susceptible to recall or reporting bias. Finally, as a cross-sectional study, causal inferences regarding the role of consanguinity or awareness in mutation transmission cannot be established. Future studies incorporating larger, multi-regional samples and comprehensive molecular and clinical evaluations are needed to better understand the genetic landscape and clinical implications of *β*-thalassemia in Pakistan.

## Conclusion

This study underscores the high prevalence and mutational complexity of *β*-thalassemia in northern Pakistan, driven primarily by the IVS I-5 and FSC 8/9 variants and reinforced by consanguineous marriages. The marked regional variation and presence of compound heterozygotes reveal a more complex genetic landscape than previously recognized, highlighting the need to expand molecular diagnostic capacity. These findings call for a dual public-health strategy: immediate implementation of targeted genetic education, premarital screening, and counseling programs, alongside sustained investment in diagnostic infrastructure, particularly in underserved rural areas. Addressing these gaps is essential to a National Thalassemia Control Program aimed at reducing disease burden and improving health outcomes across Pakistan.

## Data Availability

The datasets generated and/or analyzed during the current study are available from the corresponding author on reasonable request.

## Abbreviations

ARMS-PCR: Amplification refractory mutation system-polymerase chain reaction
CBC: Complete blood count
CI: Confidence interval
DNA: Deoxyribonucleic acid
HBB: Hemoglobin subunit beta
Hb: Hemoglobin
HbA: Adult hemoglobin A
HbA_2_: Hemoglobin A_2_
HbF: Fetal hemoglobin
PND: Prenatal diagnosis
RBC: Red blood cell
